# Hic-5 is required for activation of pancreatic stellate cells and development of pancreatic fibrosis in chronic pancreatitis

**DOI:** 10.1038/s41598-020-76095-1

**Published:** 2020-11-05

**Authors:** Lin Gao, Xiao-Feng Lei, Aya Miyauchi, Masahito Noguchi, Tomokatsu Omoto, Shogo Haraguchi, Takuro Miyazaki, Akira Miyazaki, Joo-ri Kim-Kaneyama

**Affiliations:** 1 Department of Biochemistry, Showa University School of Medicine, 1-5-8 Hatanodai, Shinagawa-ku, Tokyo, 142-8555 Japan; 2 Department of Health Management, The Affiliated Hospital of Southwest Medical University, Luzhou, China

**Keywords:** Gastrointestinal diseases, Pancreatitis

## Abstract

Accumulated evidence suggests that activated pancreatic stellate cells (PSCs) serve as the main source of the extracellular matrix proteins accumulated under the pathological conditions leading to pancreatic fibrosis in chronic pancreatitis (CP). However, little is known about the mechanisms of PSC activation. PSCs have morphologic and functional similarities to hepatic stellate cells, which are activated by hydrogen peroxide-inducible clone-5 (Hic-5), a TGF-β1-induced protein. In this study, we investigated whether Hic-5 activates PSCs, which promote pancreatic fibrosis development in CP. *Hic-5*-knockout and wild type mice were subjected to caerulein injection to induce CP. Hic-5 expression was strongly upregulated in activated PSCs from human CP tissue and from mouse pancreatic fibrosis in caerulein-induced CP. Hic-5 deficiency significantly attenuated mouse pancreatic fibrosis and PSC activation in the experimental murine CP model. Mechanistically, Hic-5 knock down significantly inhibited the TGF-β/Smad2 signaling pathway, resulting in reduced collagen production and α-smooth muscle actin expression in the activated PSCs. Taken together, we propose Hic-5 as a potential marker of activated PSCs and a novel therapeutic target in CP treatment.

## Introduction

Chronic pancreatitis (CP) is an inflammatory disease of the exocrine pancreas that increases the risk of developing pancreatic cancer. The pathological hallmarks of CP are acinar cell injury and gradual fibrosis of the pancreas, which are associated with the loss of pancreatic function. Currently, no clinical therapy is available for reversing the fibrotic damage associated with CP^1,2^.

There is accumulating evidence suggesting that activated pancreatic stellate cells (PSCs) are critical for the formation of extracellular matrix (ECM) in pancreatic fibrosis in CP^3^. In normal pancreas, PSCs are present in their quiescent phenotype and can be identified as the vitamin A-containing cells. In response to pancreatic injury or inflammation, quiescent PSCs undergo activation. Activated PSCs have increased α-smooth muscle actin (α-SMA) expression, which is an activated PSC marker, and excessive release of ECM proteins such as collagen type I and III^4^. One of the major activators of PSCs is transforming growth factor β (TGF-β), which actives the TGF-β1-dependent Smad2 profibrotic signaling in fibrotic diseases^5^. PSCs have morphological and functional similarities to hepatic stellate cells (HSCs), which play a major role in liver fibrogenesis^6^.

As we previously reported, hydrogen peroxide-inducible clone 5 (Hic-5) plays a critical role in liver fibrosis^5^. Hic-5-deficient mice show significantly reduced mouse liver fibrosis and HSC activation. Furthermore, Hic-5 enhanced the activation of HSCs by regulating TGF-β1-dependent Smad2 and *Hic-5* siRNA reduced liver fibrosis in carbon tetrachloride (CCL4)-treated mice. However, Hic-5 mutations have not been studied in the context of CP.

Hic-5, also known as TGF-β-1-induced transcript 1 (TGFβ1i1), is an adhesion scaffold protein that is expressed in the nucleus and in cell membrane adhesion plaques depending on the cell type and cell signals^7,8^. Its main function is to control the cytoskeleton^9^. Hic-5 has been reported to be essential for matrix ECM deposition and remodeling in vivo^10^.

Recent studies have shown that Hic-5 plays an important role in several fibrotic diseases, including glomerular sclerosis, intestinal fibrosis and liver fibrosis, by regulating myofibroblast differentiation and ECM proteins expression^11,12^. Hic-5 expression was positively correlated with the expression of the HSC activation marker, α-SMA, during liver fibrosis^5^. However, the mechanism underlying Hic-5-mediated regulation of CP has not been determined.

Herein, we studied CP in a mouse model mimicking the human disease and investigated the role of Hic-5 in the development of fibrosis and its potential as a therapeutic target for CP.

## Materials and methods

### Pancreatic fibrosis induction

Wild type (WT) and *Hic-5*-knockout (Hic-5 KO) mice (C57BL/6 background) were maintained under specific pathogen-free conditions in the animal care facility of Showa University School of Medicine^5^. Experiments were performed on age- and sex-matched mice at 8–12 weeks of age. CP was induced as described by Westphalen et al.^13^ with slight modification by repeated intraperitoneal injections of 50 μg/kg caerulein (Sigma-Aldrich, C9026) every hour for 6 h twice a week (Monday and Thursday) for 6 weeks. Controls received PBS injections. All experiments were approved by the regional Animal Study Committees of Showa University School of Medicine and performed according to the institutional guidelines stipulated by Showa University School of Medicine.

### Human pancreatic tissue and pancreatic stellate cells

Human Pancreatic tissue arrays were purchased from Biomax Inc (Catalog #BBS14011). Human primary PSCs were purchased from ScienCell Research Laboratories (Catalog #3830) and cultured in stellate cell medium (Catalog #5301) according to the manufacturer’s instructions.

### Cell isolation

PSCs were isolated from WT and Hic-5 KO mice, as described previously^14^. Briefly, PSCs were isolated from the pancreas by collagenase digestion and Nycodenz density gradient centrifugation. The pancreases of three mice were pooled to obtain enough cells for the experimental setting. PSCs were isolated from WT and Hic-5 KO mice at 8–12 weeks of age as described by Apte et al*.*^14^ Isolated PSCs were washed with GBSS and resuspended in DMEM (Invitrogen) containing 10% characterized fetal bovine serum (GIBCO) and antibiotics (100 U/mL penicillin and 100 mg/mL streptomycin, Invitrogen). Cells were used before the first passage.

### Real-time qPCR

Real-time PCR was performed using procedures described previously^5^. Total RNA was obtained from pancreas tissues or cultured cells using the Trizol Reagent (Invitrogen) and an ABI7900 real-time PCR detection system (Applied Biosystems, CA, USA). The expression values were normalized against a housekeeping gene (*GAPDH*), and fold changes were calculated relative to the control group using the 2^−∆∆Ct^ method. Each sample was analyzed in duplicate. The primers are listed in the previously published protocol^5^.

### Western blotting

Western blotting was performed as previously described^5^. Tissues and cells were lysed with RIPA lysis buffer. Lysates were separated by SDS-PAGE, transferred to polyvinylidene difluoride membranes (GE Healthcare, MA, USA) and blocked with 5% non-fat milk in phosphate-buffered saline (PBS) containing 0.1% Tween 20. Membranes were incubated with primary antibodies against Hic-5 (Catalog No. 611165, 1:500, BD Transduction Laboratorie), GAPDH (171-3, MBL, 1:2000, Nagoya), α-SMA (Catalog No. ab5694, 1:500, abcam), Collagen I (Catalog No. ab6308, 1:1000, abcam), Smad2 (Catalog No. #5339, 1:500, cell-signal), or p-Smad2 (Catalog No. #3108, 1:500, cell-signal). After incubation with horseradish peroxidase-conjugated secondary antibodies, positive bands were visualized using Western Lightning chemiluminescence reagent (Wako, Osaka, Japan), followed by exposure to X-ray film (Fuji Film, Tokyo, Japan). The band densities were measured using Densitograph software (ATTO, Tokyo, Japan).

### Hematoxylin and eosin staining, Masson's trichrome staining and Sirius Red staining

Tissue staining was performed as previously described^5^. Pancreatic tissues were immediately fixed in 4% paraformaldehyde for paraffin embedding. Tissue specimens were cut into 4-μm thick serial sections for hematoxylin and eosin (H&E) staining, Masson's trichrome staining and Sirius Red staining. H&E staining was performed using a commercial kit (Catalog No. C0105, Beyotime) according to the manufacturer's instructions. Masson's trichrome staining was performed with a staining kit following the manufacturer's protocol (Catalog No. G1340, Solarbio). Sirius Red staining of paraffin-embedded pancreas sections was performed with a staining kit (Catalog No. S8060, Solarbio). Fibrotic areas that appeared blue under Masson's trichrome staining and Red under Sirius Red staining were quantified by ImageJ 1.43 (W. S. Rasband, ImageJ, National Institutes of Health). The non-pancreatic regions were subtracted when calculating the total area of pancreatic tissue in the selected field. The percentage of the fibrotic area was calculated from the ratio of fibrotic tissue to total pancreatic tissue.

### Immunohistochemistry

Immunohistochemistry was performed using procedures described previously^5^. Antigen retrieval was performed by heating slides in the microwave for 20 min in 0.01 mol/L citrate buffer (pH 6.0), and 3% hydrogen peroxide was added for 10 min to quench peroxidase activity. Sections were treated with normal goat serum, followed by incubation overnight with anti-Hic-5 antibody (Catalog No. 611165, 1:100, BD Transduction Laboratorie) at 4 °C. Sections were then rinsed with PBS, incubated with secondary antibodies for 1 h, stained with diaminobenzidine and counterstained with hematoxylin. Sections were dehydrated and sealed, and then examined under a microscope.

### Immunofluorescence

The samples were processed using procedures described previously^5^. Tissues and cells were fixed in 4% paraformaldehyde in PBS for 10 min at 4 °C, permeabilized with 0.01% Triton X-100 in PBS for 10 min, and then treated with 10% BSA for 1 h at 25 °C. The cells were then incubated with rabbit anti-α-SMA (Catalog No. ab5694, 1:100, abcam) or anti-Hic-5 antibody (Catalog No. 611165, 1:100, BD Transduction Laboratorie) overnight at 4 °C. After washing with PBS to remove excess primary antibodies, cells were incubated with fluorochrome-conjugated secondary antibodies (anti-rabbit Alexa 488 for α-SMA and anti-mouse Alexa 568 for Hic-5) for 30 min. Finally, cells were stained with Hoechst 33258 (Catalog No. C1011, Beyotime) for 20 min at room temperature to visualize the nuclei.

### Statistical analysis

For statistical analysis data were analyzed with Student's *t*-test and are presented as mean ± SD of triplicate measurements in three separate experiments. Statistical analysis was performed with GraphPad Prism 6.0 software (GraphPad Software Inc., San Diego, CA, USA). *p* < 0.05 was considered statistically significant.

## Results

### Hic-5 expression is enhanced in CP and activated PSCs

**Figure 1:**
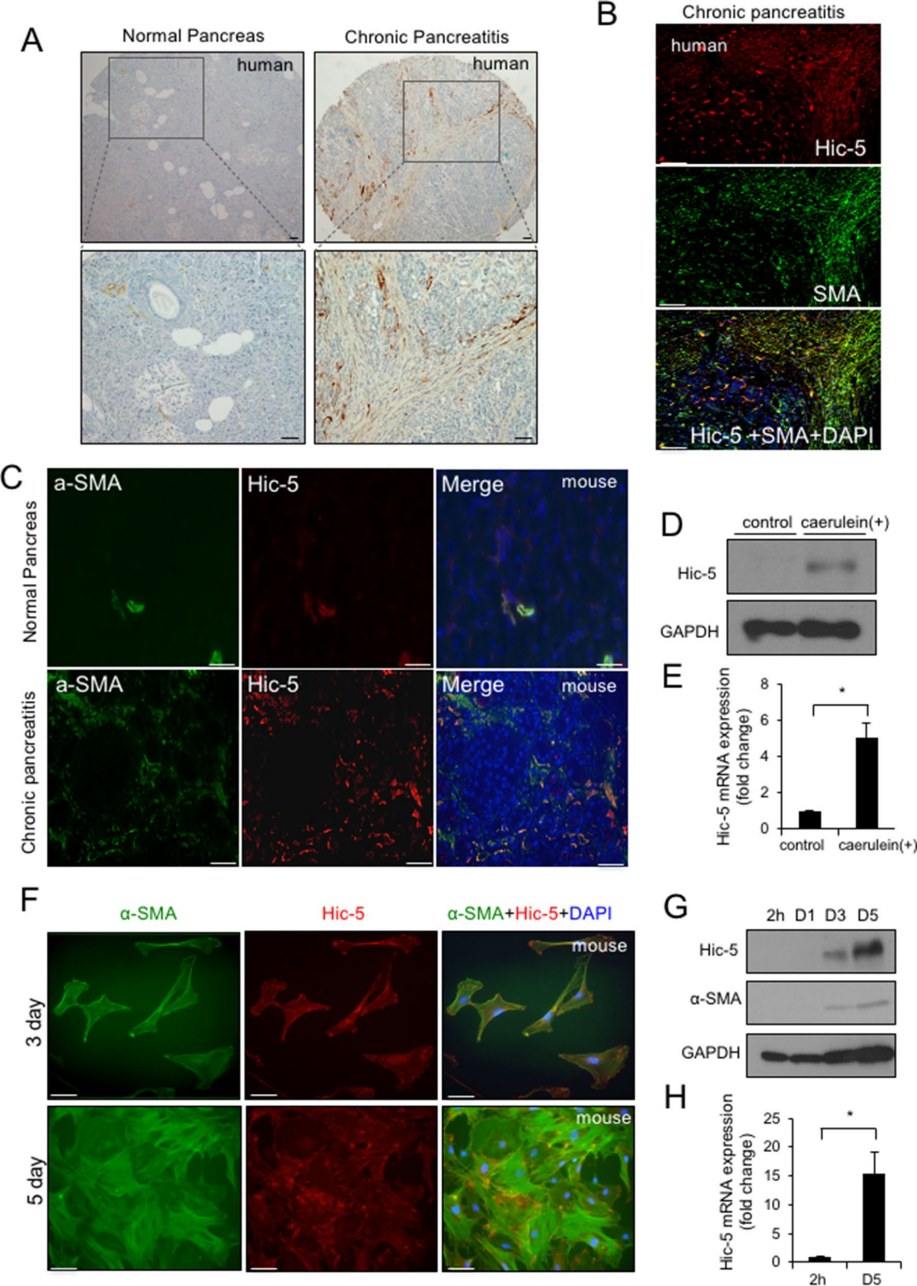
Hic-5 expression is enhanced in CP and in activated PSCs. (**A**) Immunohistochemical staining of Hic-5 in normal human pancreatic tissue (left) and chronic pancreatitis (CP) tissue (right); magnification, × 100 (top), × 400 (bottom). (**B**) Double immunofluorescence staining of Hic-5 (red) and α-SMA (green) in human pancreatic tissue with CP; nucleus (blue). Scale bars indicate 50 μm (**C**) Double immunofluorescence staining of Hic-5 (red) and α-SMA (green) in mouse pancreatic tissue with CP; nucleus (blue). Scale bars indicate 50 μm (**D**) Western blot analysis of Hic-5 expression in mouse pancreas from WT mice with or without caerulein treatment. (**E**) mRNA expression of *Hic-5* in mouse pancreas with or without caerulein-treatment. (**F**) Double immunofluorescence staining of Hic-5 (red) and α-SMA (green) in cultured PSCs from WT mice at the indicated time points. Scale bars indicate 20 μm (**G**) Western blot analysis of Hic-5 and α-SMA expression in cultured PSCs from WT mice at the indicated time points. (**H**) mRNA expression of *Hic-5* in cultured PSCs from WT mice at the indicated time points. *p < 0.05.

To evaluate Hic-5 expression levels in human pancreatic tissues, we performed immunohistochemistry on normal pancreas and CP tissues. In normal pancreas, Hic-5 expression was only found in vascular smooth muscle cells of stromal tissues. However, in CP tissues, significantly enhanced expression of Hic-5 was observed in spindle-shaped stromal cells that resemble the morphologic characteristics of activated PSCs (Fig. 1A). We also performed an immunofluorescence staining of Hic-5 and α-SMA, which is a marker of activated PSCs. We found that almost all of the cells that were positive for Hic-5 also expressed α-SMA (Fig. 1B). These findings suggest that Hic-5 is expressed in activated PSCs in human CP tissues. We then analyzed Hic-5 levels in a murine model of caerulein-induced CP. Similar to the human results, immunofluorescence microscopy revealed colocalization of Hic-5 and α-SMA in the CP model, suggesting that Hic-5 is expressed in activated moues PSCs (Fig. 1C). Western blotting and qPCR analysis showed a significant increase in Hic-5 expression after caerulein injection compared with the controls (Fig. 1D,E). We next analyzed the expression of Hic-5 and α-SMA in isolated primary mouse PSCs undergoing differentiation from quiescent to activated PSCs at different time points (from 2 h to 5 days) by immunofluorescence, western blotting and qPCR analyses (Fig. 1F–H). Hic-5 was detected at focal adhesions of cells at day 3 and day 5 of culture. Both Hic-5 and α-SMA were not detected in quiescent PSCs (2 h and day 1 of culture), however they were significantly upregulated in activated PSCs (days 3 and 5 of culture), suggesting that Hic-5 is a potential marker of activated PSCs.

### Hic-5 deficiency reduces fibrosis development in caerulein-induced CP

**Figure 2:**
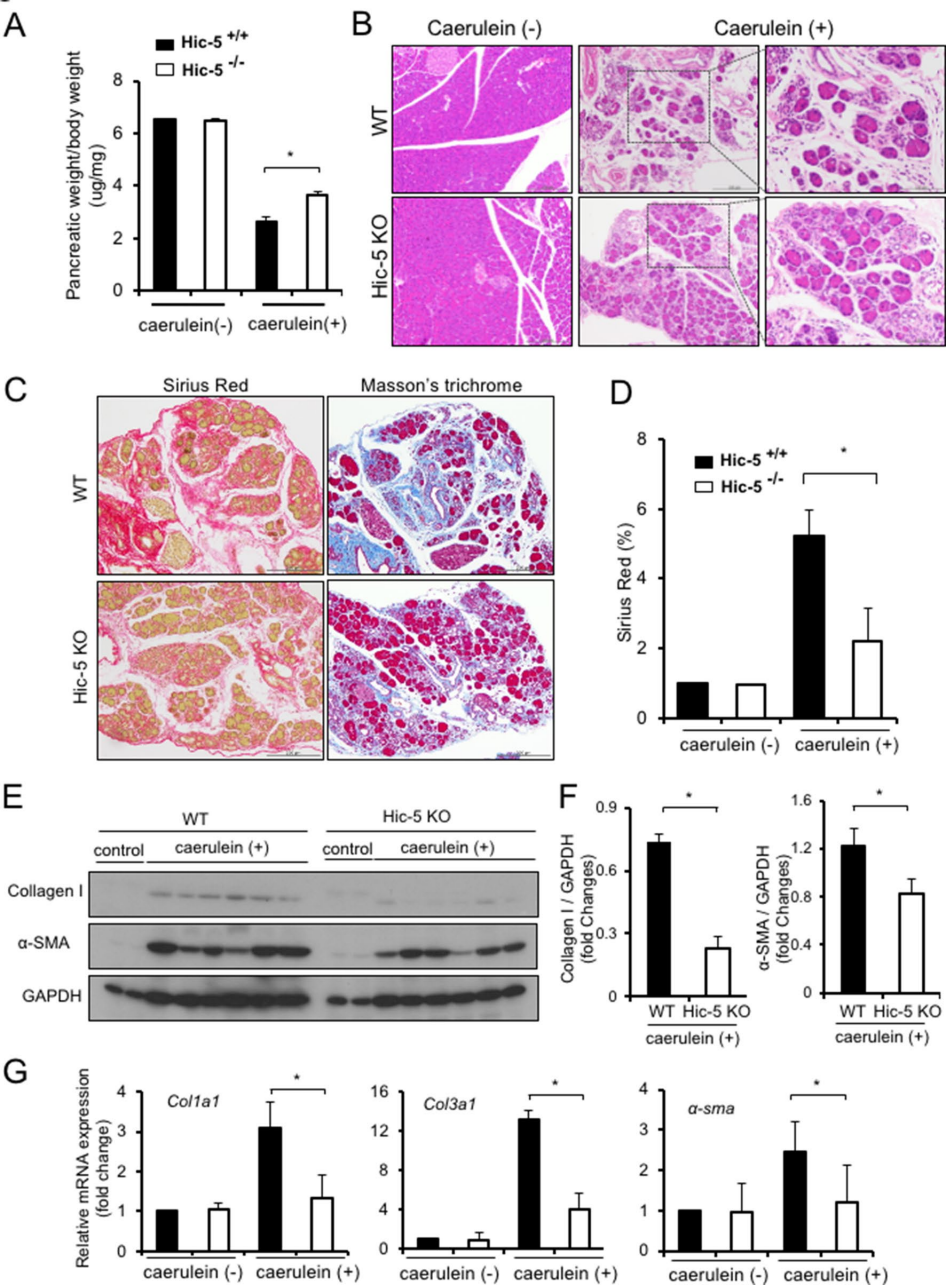
Hic-5 deficiency reduces fibrosis development in caerulein-induced CP. (**A**) Determination of the ratio of pancreatic tissue weight to mouse body weight. (**B**) Mouse pancreatic tissues examined by H&E staining. WT (top left × 100), Hic-5 KO (bottom left × 100), WT CP (upper × 100, top right × 400) and Hic-5 KO CP (lower × 100, lower right × 400). (**C**) Sirius Red staining and Masson’s trichrome staining of mouse pancreatic tissue. (**D**) Determination of the ratio of the Sirius Red stained area to total tissue size. (**E**) The expression of Collagen I and α-SMA was detected by western blotting. (**F**) Quantitative analysis of Collagen I and α-SMA expression (E) after normalization against GAPDH. (**G**) mRNA analysis of *Col1a1*, *Col3a1*, and α*-sma* expression. *p < 0.05.

To investigate the roles of Hic-5 in CP, we induced CP using caerulein injection in WT and Hic-5 KO mice. After treating mice with caerulein, we found that the pancreatic shrinkage in Hic-5 KO mice was significantly attenuated compared with WT mice (Fig. 2A). H&E, Sirius Red and Masson’s trichrome staining assays were used for histological analysis of CP. Compared with WT mice, we found significant reduction in acinar cell loss and in pancreatic fibrosis in Hic-5 KO mice (Fig. 2B–D, Supplementary Fig. S1). We also assessed the protein levels of α-SMA in CP by western blot analysis. WT mice injected with caerulein showed high levels of α-SMA expression, however this expression was markedly reduced in Hic-5 KO mice (Fig. 2E,F). Moreover, qPCR analysis confirmed significantly lower expression of fibrosis-related genes, including collagen I (*Col1a1*) and collagen III (*Col3:1*), in Hic-5 KO mouse pancreas compared with WT mouse pancreas after caerulein injection (Fig. 2G). Interestingly, there was no difference in *TGF-β1* mRNA expressions between WT and Hic-5 KO mice (data not shown). These results suggest that Hic-5 contributes to the development of fibrosis in caerulein-induced CP.

### Hic-5 deficiency attenuates activation of cultured mouse PSCs

**Figure 3:**
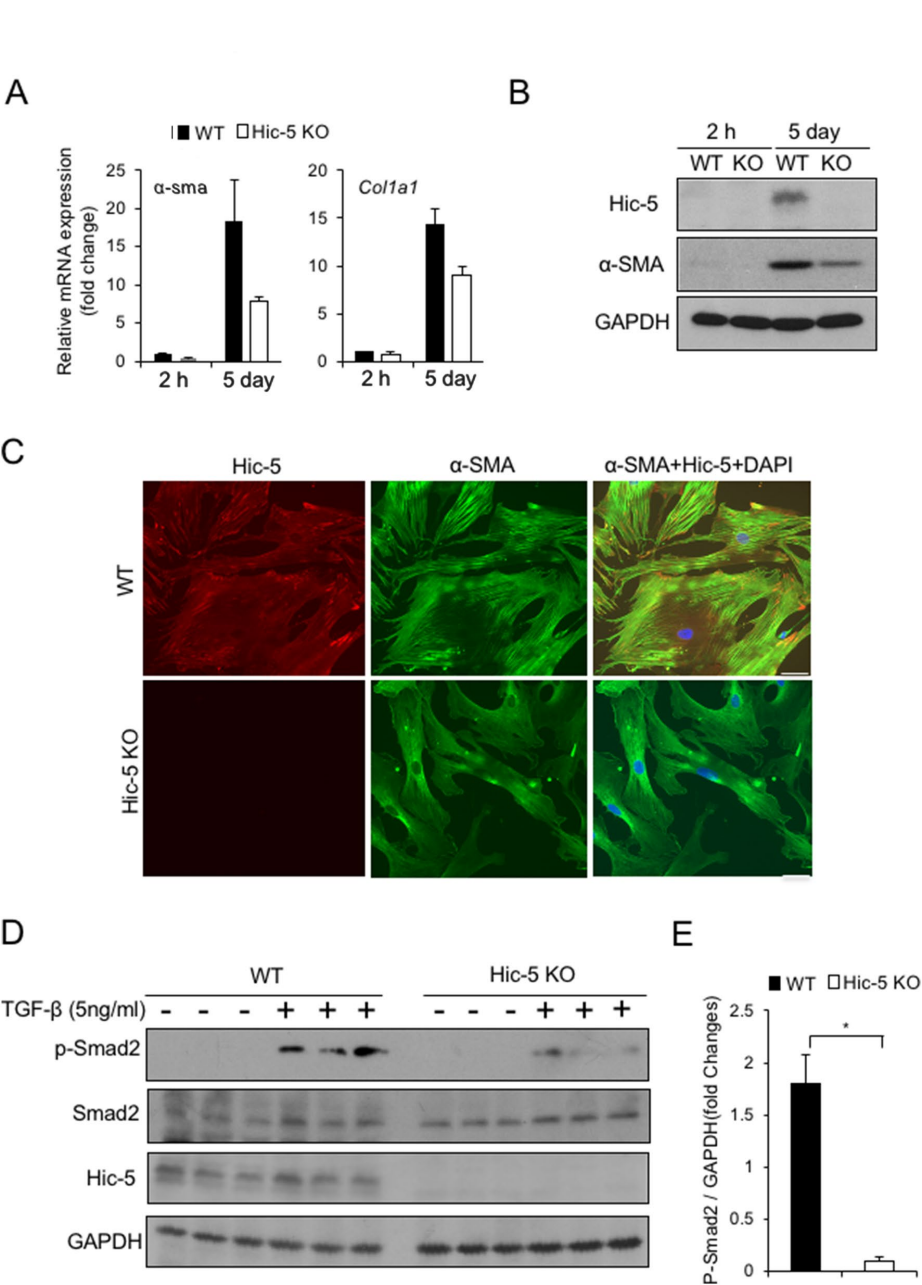
Hic-5 deficiency attenuates activation of cultured mouse PSCs by impairing Smad2 phosphorylation. (**A**) The expression of *Col1a1* and α-*SMA* mRNA in PSCs from the two groups was detected by RT-qPCR. (**B**) The expression of Hic-5 and α-SMA in PSCs from the two groups was detected by western blotting at 2 h and at 5 days. (**C**) Detection of Hic-5 (red) and α-SMA (green) expression by immunofluorescence. The upper panels show PSCs isolated from WT mice. The lower panels show PSCs isolated from Hic-5 KO mice. Scale bars indicate 20 μm (**D**) The expression of p-Smad2 and Hic-5 was detected by western blotting. (**E**) Quantitative analysis of p-Smad2 is shown. *p < 0.05.

PSCs are in their quiescent phenotype in the healthy pancreas; when activated, PSCs are transformed into myofibroblast-like cells. We speculated that Hic-5 mediates PSC activation during the transition from the quiescent to myofibroblast-like phenotype. To evaluate the role of Hic-5 in PSC activation, we isolated PSCs from the pancreases of WT and Hic-5 KO mice and cultured them for 5 days. WT PSCs at day 5 switched to myofibroblast-like cells characterized by loss of vitamin A lipid droplets and increased α-SMA expression (Fig. 1G). qPCR analysis showed a significant decrease in the expression of both α-SMA and collagen I, genes associated with activated PSCs, in Hic-5 KO PSCs compared with WT PSCs (Fig. 3A). In addition, the protein level of α-SMA was reduced in Hic-5 KO PSCs (Fig. 3B). Consistent with the mRNA levels in mouse pancreas tissues, WT and Hic-5 KO PSCs activated in culture had similar *TGF-β1* mRNA expression (data not shown). Also, Hic-5 deficiency did not affect inflammatory cytokine levels in activated PSCs (data not shown). Furthermore, microscopy observation of cells stained with α-SMA and Hic-5 showed myofibroblast-like cell differentiation in WT PSCs, however, these morphological changes were attenuated in Hic-5 KO PSCs (Fig. 3C). These results indicate that the activation of mouse PSCs is mediated through the expression of Hic-5 and imply that Hic-5 serves as a potential novel activation marker for mouse PSCs.

### Hic-5 deficiency impairs Smad2 phosphorylation in cultured murine PSCs

The TGF-β/Smad pathway has previously been shown to promote PSC activation and ECM production. Studies have revealed that TGF-β1 stimulated PSC activation through a Smad2-dependent pathway^15,16^. We sought to determine the contribution of Hic-5 to the activation of the TGF-β/Smad pathway. By using PSCs isolated from WT and Hic-5 KO mice, we found no difference in Smad2 expression between WT PSCs and Hic-5 KO PSCs. However, the phosphorylated Smad2 levels were significantly increased after TGF-β treatment in WT PSCs, but not in Hic-5 KO PSCs (Fig. 3D,E). These data suggest that the intracellular signaling pathway through which TGF-β/Smad regulates PSC functions is Hic-5-dependent.

### In vitro Hic-5 knock down attenuates activation and fibrotic function of cultured human PSCs

**Figure 4:**
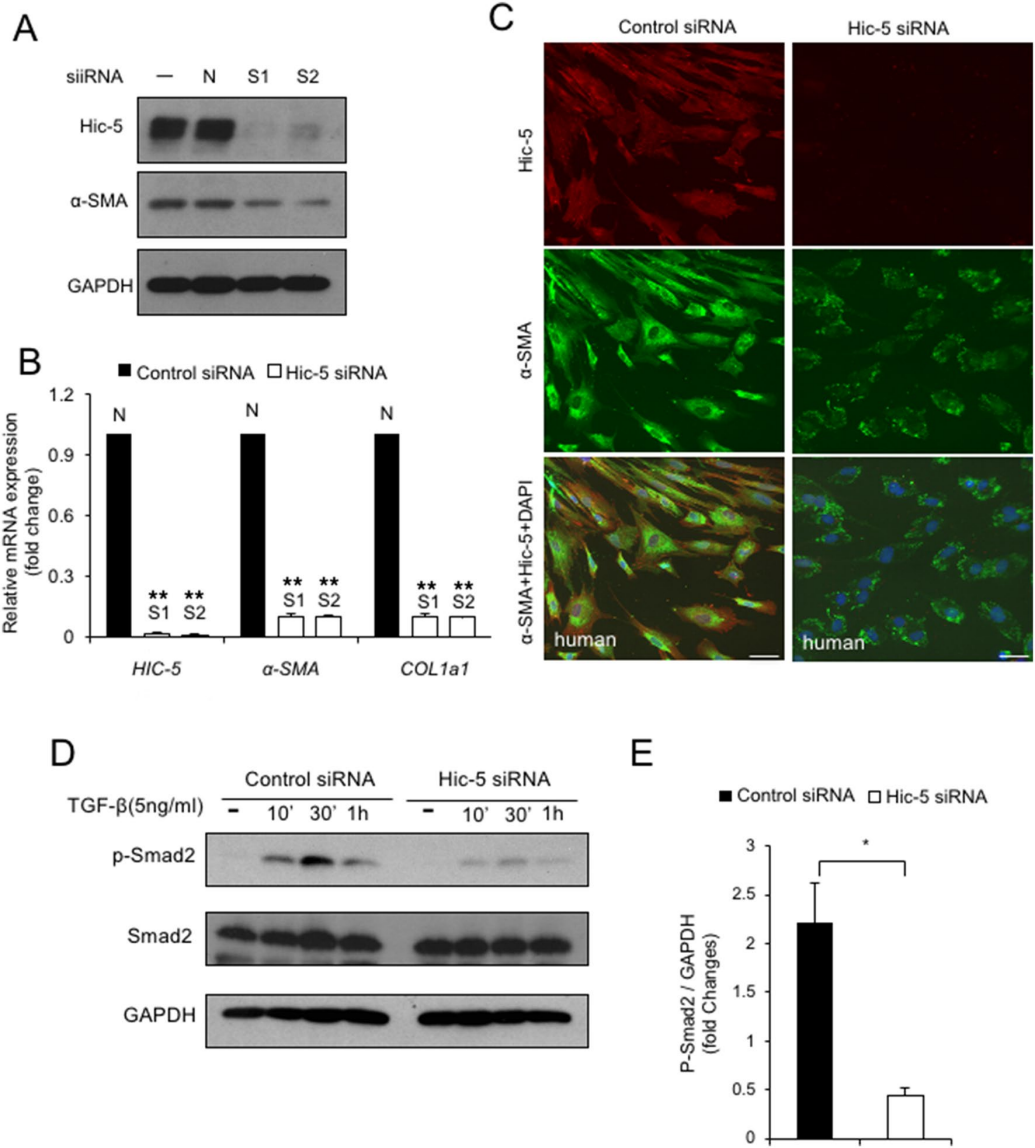
Hic-5 knockdown attenuates activation of cultured human PSCs by impairing Smad2 phosphorylation. (**A**) The expression of Hic-5 and α-SMA was examined by western blot analysis. N: control siRNA. S1 and S2: hic-5 siRNAs. (**B**) Comparison of Hic-5, α-SMA and COL1a1 relative mRNA expression in the control and hic-5 siRNA transfection group. (**C**) siRNA was transfected into human PSCs. The expression of Hic-5 (red) and α-SMA (green) was examined by immunofluorescence. Scale bars indicate 20 μm (**D**) Representative western blot analysis of p-Smad2, Smad2 and Hic-5 expression in cultured human PSCs after transfection with control siRNA or Hic-5 siRNA. (**E**) Quantitative analysis of p-Smad2. **p < 0.01, *p < 0.05.

A number of studies have suggested that activated PSCs play a crucial role in pancreatic fibrosis in CP^17,18^. To evaluate Hic-5 as a therapeutic target in CP, we knocked down Hic-5 in cultured human PSCs using two different *Hic-5* siRNAs. The efficiency of Hic-5 knockdown was assessed by western blotting. *Hic-5* siRNA treatment caused a significant decrease in Hic-5 protein expression (Fig. 4A). To analyze the role of Hic-5 in cultured human PSCs, we measured α-SMA and collagen I contents. Knockdown of Hic-5 by siRNA reduced the α-SMA and collagen I mRNA expression in PSCs relative to the controls (Fig. 4B). Furthermore, the level of α-SMA protein expression was significantly lower in *Hic-5* siRNA-treated human PSCs than in the controls (Fig. 4A). Interestingly, *Hic-5* siRNA also affected the activation morphological change in cultured human PSCs (Fig. 4C). This phenotypic difference is supported by a previous report that an amoeboid phenotype was induced by Hic-5 knockdown^19^. We next investigated whether Hic-5 contributes to the activation of the TGF-β/Smad pathway in human PSCs. The phosphorylated Smad2 levels were significantly decreased after TGF-β treatment in Hic-5-knockdown cells compared with the control siRNA (Fig. 4D,E). Taken together, these results demonstrated that *Hic-5* siRNA inhibited the activation of human cultured PSCs, proposing Hic-5 as a potential therapeutic target for pancreatic fibrosis in CP.

## Discussion

The results of the current study demonstrated that Hic-5 expression was significantly increased both in the mouse model of CP and in activated PSCs. Hic-5 knockout attenuated the activation of PSCs, and the CP severity significantly decreased in the experimental murine CP model. Furthermore, siRNA knock down of Hic-5 in human PSCs suppressed their activation. Hic-5 deficiency in both mouse and human PSCs inhibited Smad2 phosphorylation. This study demonstrated that Hic-5 deficiency slows the progression of CP by inhibiting PSC activation.

In a normal pancreas, the quiescent type of PSCs are rich in vitamin A-containing lipid droplets, and secrete only a small amount of ECM components^20^. PSCs are activated during CP as demonstrated by their fibroblast-like morphology. Additionally, the lipid droplets are decreased and the secretion of ECM components is increased, which leads to exacerbation of pancreatic fibrosis^4,21^. Therefore, activation of PSCs is considered a key event for pancreatic fibrosis^3,22^.

Because PSCs promote inflammatory and fibrotic processes during CP, they have become a major target in CP treatment strategies. Multiple studies have shown that therapies that target PSCs can reduce the severity of CP. Xiao et al*.* have demonstrated that retinoic acid treatment can reduce disease severity by reducing PSC activation in a CP mouse model^23^. Zhang et al. have demonstrated that PTEN can reduce PSC activation and pancreatic fibrosis in a rat CP model^24^. Our findings provide new evidence for attenuation of CP-related pathological changes by reducing PSC activity.

PSCs have been considered the pancreatic counterparts of HSCs^6^. Consistent with previous findings, we found in mouse and human PSCs that loss or suppressed Hic-5 expression inhibited Smad2 phosphorylation and PSC activation. These findings indicate that Hic-5 deficiency under TGF-β stimulation slowed the CP process by inhibiting the Smad2 phosphorylation. The TGF-β1/Smad signaling pathway is currently considered to be the main pathway in fibrosis formation such as renal fibrosis, liver fibrosis and pulmonary fibrosis^25,26^. Our research showed that this pathway plays an important role in fibrosis development in CP.

In addition to the deposition of collagens, the disbalance between MMPs and TIMPs is a principal feature of pancreatic fibrosis. Either protein family is responsible for both fibrogenesis and fibrolysis and are considered as putative therapeutic targets. Although we investigated whether Hic-5 is related to the regulation of these molecules, Hic-5 deficiency did not affect the MMP-2, MMP-9 and TIMP-1 expression levels in activated PSCs at least (data not shown). On the other hand, our previous study underlined the anti-fibrotic therapeutic potential of Hic-5 in liver fibrosis. In vivo Hic-5 knockdown, even after induction of liver fibrosis by CCL4, could improve the progression of pathology. From a clinical perspective, if an effective delivery system of siRNA to the pancreas would be developed, it would be possible to test whether Hic-5 inhibition could improve established fibrosis or not.

In conclusion, the present study found that Hic-5 deficiency inhibited PSC activation and CP progression by inhibiting Smad2 phosphorylation. These findings provide a potential experimental basis for the treatment of CP by targeting Hic-5.

## Supplementary information

Supplementary Figures.

## References

[1] Zhao Q, Manohar M, Wei Y, Pandol SJ, Habtezion A (2019). STING signalling protects against chronic pancreatitis by modulating Th17 response. Gut.

[2] Xu M (2016). Scoparone protects against pancreatic fibrosis via TGF-beta/Smad signaling in rats. Cell Physiol. Biochem..

[3] Tang D (2018). Galectin-1 expression in activated pancreatic satellite cells promotes fibrosis in chronic pancreatitis/pancreatic cancer via the TGF-beta1/Smad pathway. Oncol. Rep..

[4] Lunova M (2017). Hepcidin knockout mice spontaneously develop chronic pancreatitis owing to cytoplasmic iron overload in acinar cells. J. Pathol..

[5] Lei XF (2016). Hic-5 deficiency attenuates the activation of hepatic stellate cells and liver fibrosis through upregulation of Smad7 in mice. J. Hepatol..

[6] Yamamoto G (2017). Pancreatic stellate cells have distinct characteristics from hepatic stellate cells and are not the unique origin of collagen-producing cells in the pancreas. Pancreas.

[7] Shibanuma M, Mashimo J, Kuroki T, Nose K (1994). Characterization of the TGF beta 1-inducible hic-5 gene that encodes a putative novel zinc finger protein and its possible involvement in cellular senescence. J. Biol. Chem..

[8] Shibanuma M, Kim-Kaneyama JR, Sato S, Nose K (2004). A LIM protein, Hic-5, functions as a potential coactivator for Sp1. J. Cell Biochem..

[9] Nakao A (1999). Transient gene transfer and expression of Smad7 prevents bleomycin-induced lung fibrosis in mice. J. Clin. Investig..

[10] Goreczny GJ, Forsythe IJ, Turner CE (2018). Hic-5 regulates fibrillar adhesion formation to control tumor extracellular matrix remodeling through interaction with tensin1. Oncogene.

[11] Hornigold N (2010). Upregulation of Hic-5 in glomerulosclerosis and its regulation of mesangial cell apoptosis. Kidney Int..

[12] Paul J (2018). IL-17-driven intestinal fibrosis is inhibited by Itch-mediated ubiquitination of HIC-5. Mucosal Immunol..

[13] Westphalen CB (2016). Dclk1 defines quiescent pancreatic progenitors that promote injury-induced regeneration and tumorigenesis. Cell Stem Cell.

[14] Apte MV (1998). Periacinar stellate shaped cells in rat pancreas: Identification, isolation, and culture. Gut.

[15] Ohnishi H (2004). Distinct roles of Smad2-, Smad3-, and ERK-dependent pathways in transforming growth factor-beta1 regulation of pancreatic stellate cellular functions. J. Biol. Chem..

[16] Aoki H (2007). Cyclooxygenase-2 is required for activated pancreatic stellate cells to respond to proinflammatory cytokines. Am. J. Physiol. Cell Physiol..

[17] Shimizu K (2008). Pancreatic stellate cells: Molecular mechanism of pancreatic fibrosis. J. Gastroenterol. Hepatol..

[18] Erkan M (2012). StellaTUM: Current consensus and discussion on pancreatic stellate cell research. Gut.

[19] Deakin NO, Turner CE (2011). Distinct roles for paxillin and Hic-5 in regulating breast cancer cell morphology, invasion, and metastasis. Mol. Biol. Cell.

[20] Habisch H, Zhou S, Siech M, Bachem MG (2010). Interaction of stellate cells with pancreatic carcinoma cells. Cancers (Basel).

[21] Grabliauskaite K (2016). Inactivation of TGFbeta receptor II signalling in pancreatic epithelial cells promotes acinar cell proliferation, acinar-to-ductal metaplasia and fibrosis during pancreatitis. J. Pathol..

[22] Xue R (2018). A rising star in pancreatic diseases: Pancreatic stellate cells. Front. Physiol..

[23] Xiao W (2015). Retinoic acid ameliorates pancreatic fibrosis and inhibits the activation of pancreatic stellate cells in mice with experimental chronic pancreatitis via suppressing the Wnt/beta-catenin signaling pathway. PLoS ONE.

[24] Zhang X (2018). Effects of the tumor suppressor PTEN on biological behaviors of activated pancreatic stellate cells in pancreatic fibrosis. Exp. Cell Res..

[25] Chen L (2018). Central role of dysregulation of TGF-beta/Smad in CKD progression and potential targets of its treatment. Biomed. Pharmacother..

[26] Katz LH (2016). TGF-beta signaling in liver and gastrointestinal cancers. Cancer Lett..

