# Supplementary material for: Hic-5 is required for activation of pancreatic stellate cells and development of pancreatic fibrosis in chronic pancreatitis
Source: Sci Rep. 2020 Nov 5;10:19105. doi: 10.1038/s41598-020-76095-1 (PMC7645689; doi:10.1038/s41598-020-76095-1)
Supplement: Supplementary file 1 — Supplementary Figures. [file 41598_2020_76095_MOESM1_ESM.pdf]

SUPPLEMENTAL MATERIAL

Hic-5 is required for activation of pancreatic stellate cells and development of pancreatic fibrosis in chronic pancreatitis

Lin Gao[1,2], Xiao-Feng Lei[1], Aya Miyauchi[1], Masahito Noguchi[1], Tomokatsu Omoto[1], Shogo Haraguchi[1], Takuro Miyazaki[1], Akira Miyazaki[1] and Joo-ri Kim-Kaneyama[1]

[1]Department of Biochemistry, Showa University School of Medicine, Tokyo, Japan
[2]Department of Health Management, The Affiliated Hospital of Southwest Medical University, Luzhou, China

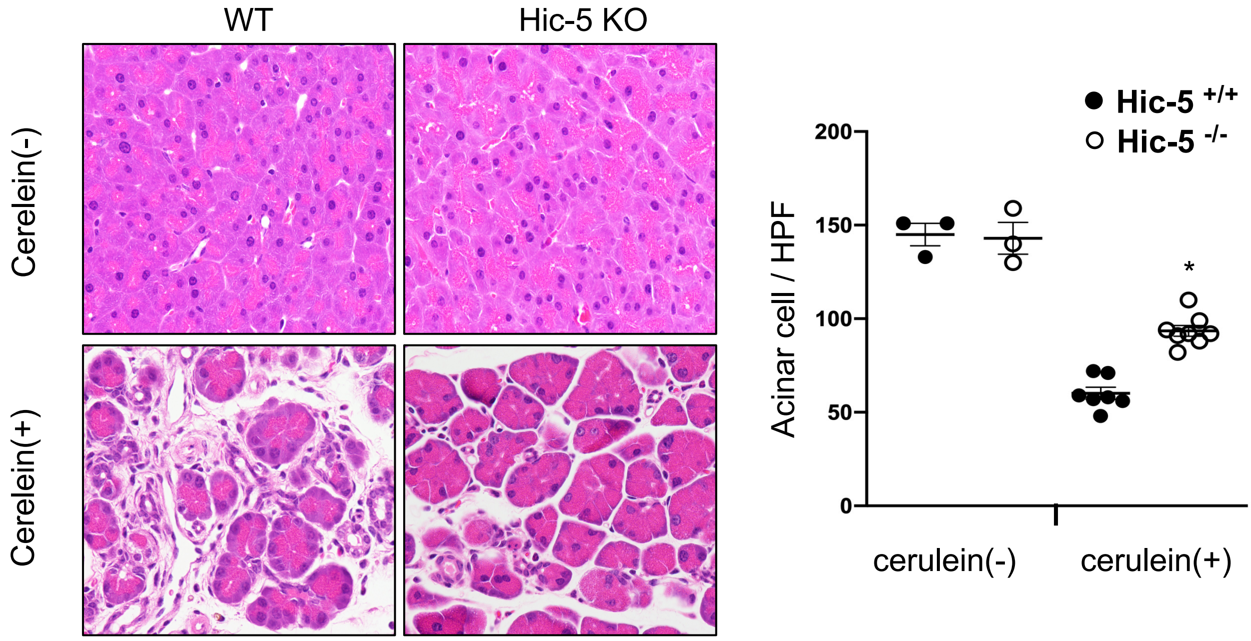

Supplementary Figure S1. Hic-5 deficiency reduces acinar cell loss in caerulein-induced CP Acinar cell loss were analyzed on HE-stained sections using a minimum of five high-power fields/slide (x400) for each of slide from mouse pancreatic tissue (seven mice/experiment for each of two independent experiments), in a blinded manner. Acinar cell were accounted with using ImageJ by a pathologist. Each bar is the mean SE. *$p<0.001$

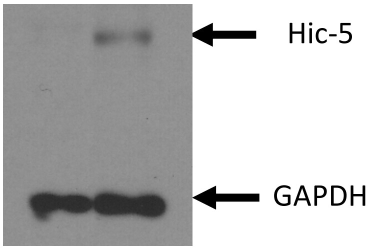

Supplementary Figure S2. Uncropped scan of Western blots (Fig1.D)

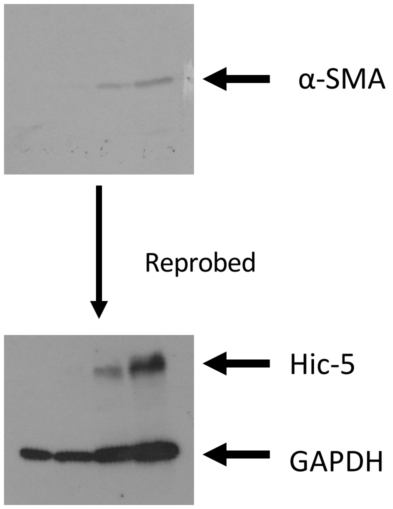

Supplementary Figure S3. Uncropped scan of Western blots (Fig1.G)

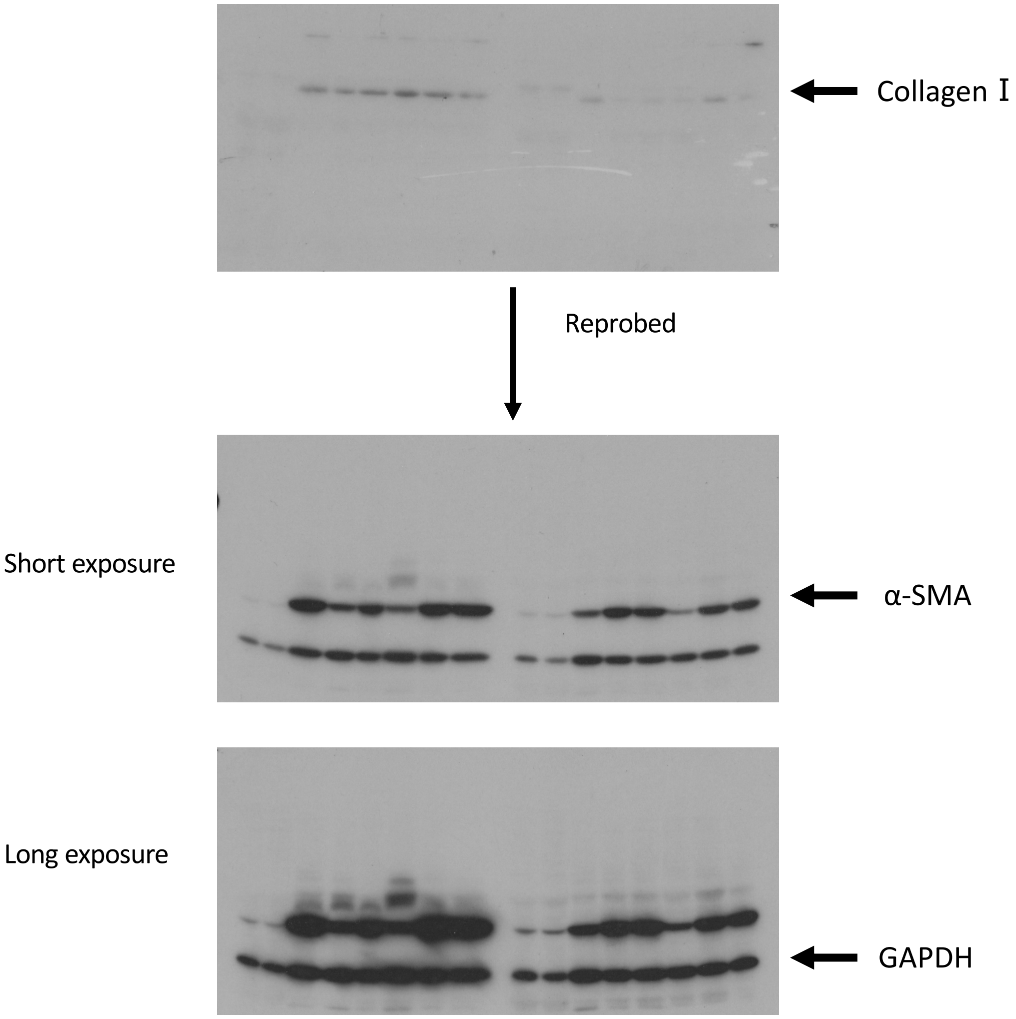

Supplementary Figure S4. Uncropped scan of Western blots (Fig2.E)

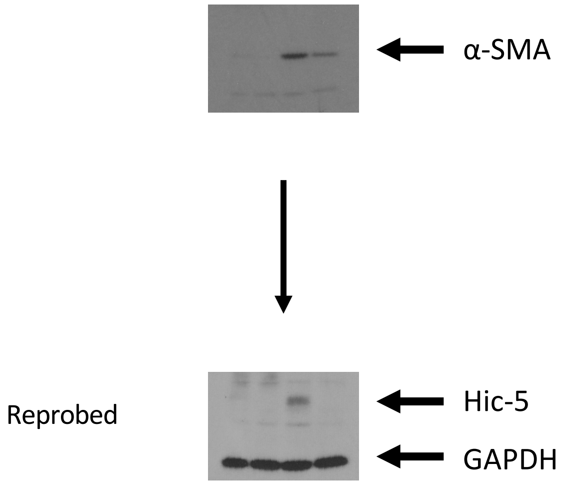

Supplementary Figure S5. Uncropped scan of Western blots (Fig3.B)

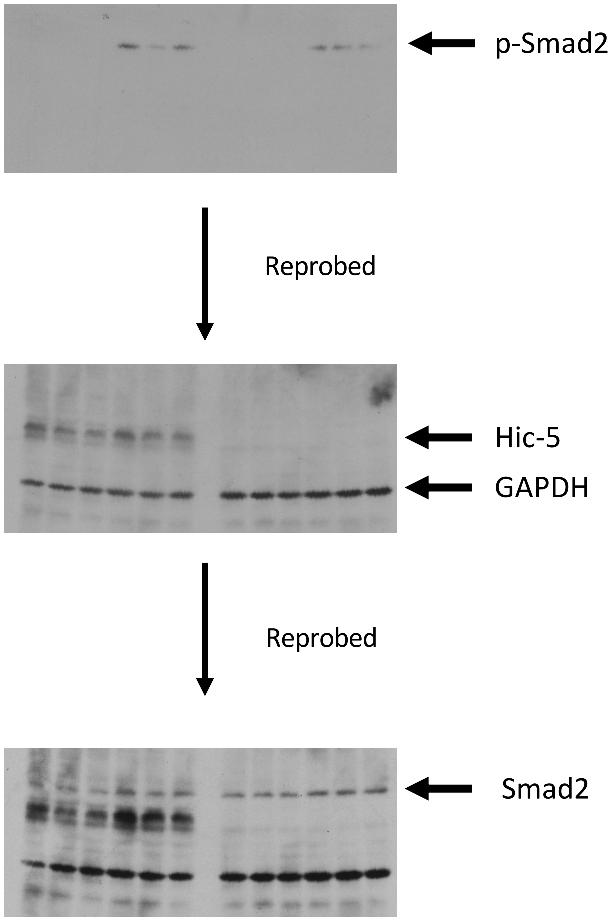

Supplementary Figure S6. Uncropped scan of Western blots (Fig3.D)

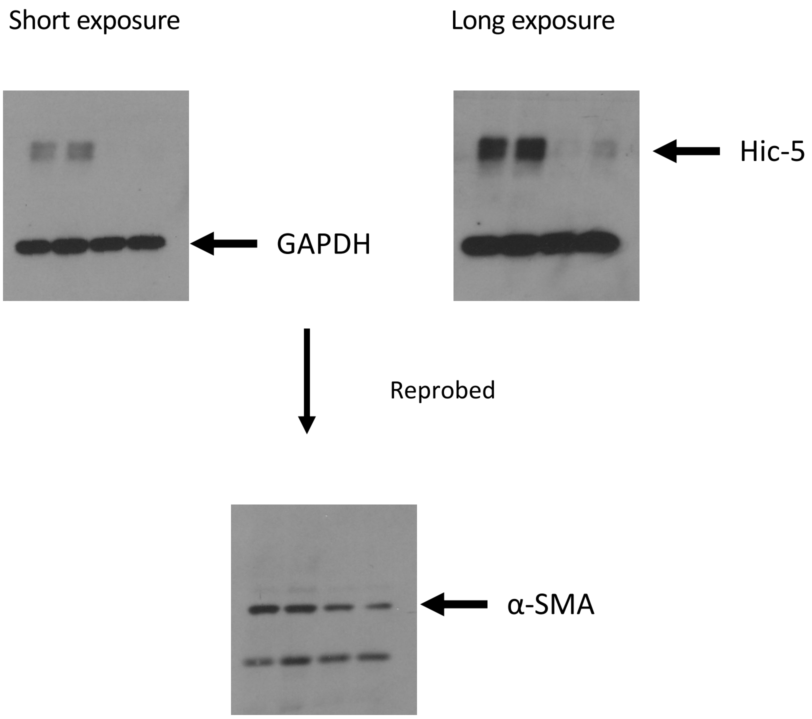

Supplementary Figure S7. Uncropped scan of Western blots (Fig4.A)

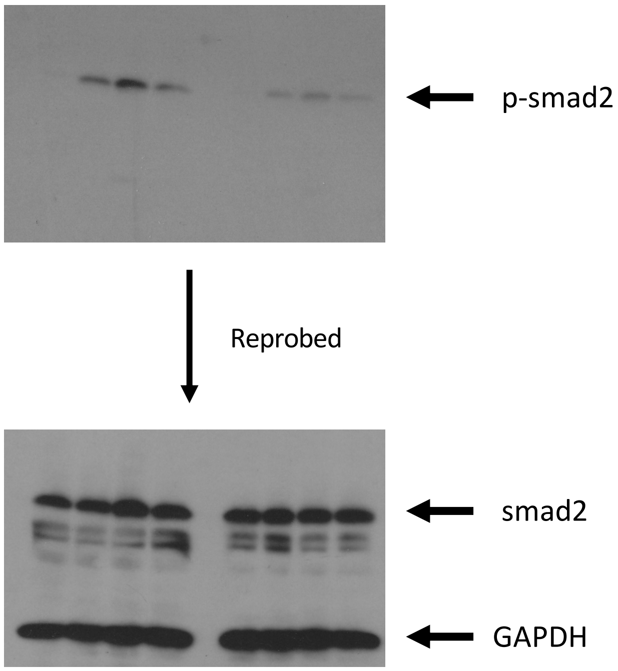

Supplementary Figure S8. Uncropped scan of Western blots (Fig4.D)

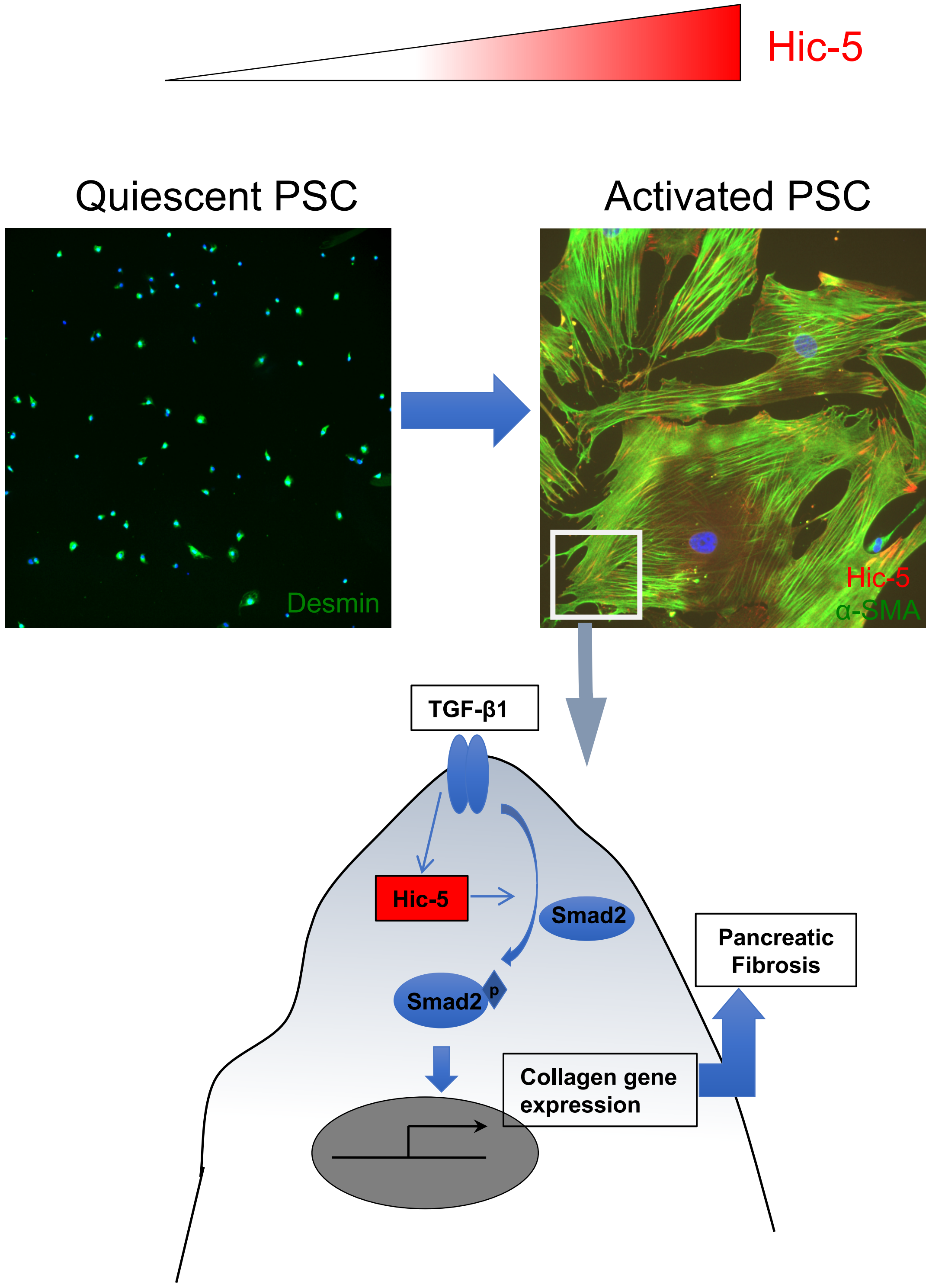

Supplementary Figure S9 A signal transduction diagram showing that Hic-5 enhances TGF-β signaling in chronic pancreatitis. As a conclusion, our study identified Hic-5 as a novel regulator of chronic pancreatitis through activation of PSCs during pancreatic injury. Hic-5 is considered a potential marker of activated PSCs and therapeutic target in the treatment of chronic pancreatitis.
